# Ultra-fast light-field microscopy with event detection

**DOI:** 10.1038/s41377-024-01603-1

**Published:** 2024-11-07

**Authors:** Liheng Bian, Xuyang Chang, Hanwen Xu, Jun Zhang

**Affiliations:** State Key Laboratory of CNS/ATM & MIIT Key Laboratory of Complex-field Intelligent Sensing, Beijing Institute of Technology, No 5 Zhongguancun South Street, Haidian District, 100081 Beijing, China

**Keywords:** Microscopy, Imaging and sensing, Ultrafast photonics

## Abstract

The event detection technique has been introduced to light-field microscopy, boosting its imaging speed in orders of magnitude with simultaneous axial resolution enhancement in scattering medium.

As lift science has rapidly developed in the last decades, the demand for novel optical instruments is becoming increasingly urgent, helping discover new biomedical phenomena and mechanisms with novel optical imaging functions regarding information dimension, imaging resolution, acquisition speed, and so on. In this regard, the light-field microscopy (LFM) technique has arisen as a novel imaging tool to acquire not only spatial but also angular information of specimens, thus realizing volumetric imaging that can reveal axial depth information for useful perspective views and focal stacks^1^. The general working principle of LFM is placing a microlens array at either the image or Fourier plane, enabling to encode angular information of different regions into a snapshot. The volumetric data cube can then be recovered from the measurement using reconstruction algorithms. Such a representative computational imaging modality maintains 3D imaging and aberration correction abilities, and has been widely applied in neuronal activity recording^2^, subcellular interaction observation^3^, turbulence-corrected telescope^4^, and so on.

As the spatial and angular resolution of LFM have been improved in recent years using high-resolution image sensors, advanced optics design, and cutting-edge deep-learning algorithms^5–8^, the imaging speed is still a remaining challenge that is directly limited by the camera’s acquisition speed^9,10^. Even using high-speed cameras, the output large number of frames put a great load on system bandwidth and post-processing algorithms. Further, the decreased exposure time of high-speed acquisition may increase measurement noise that degrades imaging quality. In this sense, the development of LFM falls into the tradeoff among imaging resolution, imaging speed, and processing efficiency under limited throughput and computing power.

To break the imaging speed limitation, in a newly published paper^11^ in *Light: Science & Applications*, Ruipeng Guo, Qianwan Yang, Andrew S. Chang, Guorong Hu, Joseph Greene, Christopher V. Gabel, Lei Tian from Boston University, and Sixian You from Massachusetts Institute of Technology, have reported an EventLFM technique that realized ultra-fast light-field imaging at kHz frame rates (Fig. 1). Different from the conventional LFM principle that directly captures light brightness, the EventLFM technique introduces an event camera to only acquire brightness variation instead, thus enabling transient feature acquisition and simultaneous data amount reduction, bypassing the limitations of low frame rate and large data amount. The authors have also developed a deep-learning-based reconstruction algorithm to recover 3D dynamics from the snapshot event measurement. Experiments demonstrate successful reconstruction of fast-moving and rapidly blinking 3D fluorescent samples at kHz frame rates. With such an event acquisition modality, the technique naturally maintains anti-scattering ability due to free of intensity accumulation in scattering medium, and has demonstrated blinking neuronal signal recording in scattering mouse brain tissues and 3D tracking of GFP-labeled neurons in freely moving *C. elegans*.

**Fig. 1:**
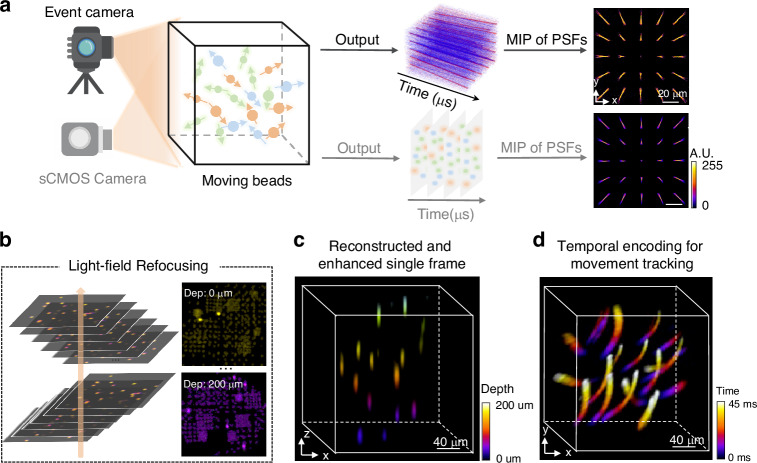
Overview of introducing event detection into light-field microscopy^11^. **a** Comparison of event camera and sCMOS camera for light-field microscopy. **b** Event stream is utilized to generate time-surface frame with the corresponding algorithm, then each time-surface frame is reconstructed using a light-field refocusing algorithm. **c** Color-coded 3D light-field reconstruction of the object with an optional deep-learning technique. **d** Color-coded 3D motion trajectory reconstructed over a 45 ms time span. The subfigures are cropped from the corresponding reference

This work initiates a novel research perspective for high-speed and anti-scattering imaging in microscopy. Besides, the event detection technique can also be incorporated into different computational imaging systems to enhance imaging speed, such as snapshot compressive imaging^12^, ptychographic imaging^13^, and so on. Along with the event detection mechanism, as the encoding process is regarding brightness variation, novel decoding algorithms are correspondingly necessary with different-format measurements, and also with special attention on measurement noise that may be more serious than intensity detection.

Following the heuristic idea of introducing novel detection devices into existing imaging systems for performance enhancement, the research perspective can be expanded to a variety of different cutting-edge image sensors and devices. Regarding detection sensitivity, the emerging single-photon detector^14^, spiking camera^15^, quantum detection system^16^ and corresponding processing techniques^17^ can replace the conventional CMOS or CCD detectors for unprecedented sensitivity of weak signals (Fig. 2). For imaging resolution, the advanced gigapixel camera^18^ can provide more pixels to reveal fine details. Concerning information dimension, high-dimensional detectors can be introduced to acquire more information of spectrum^19,20^, phase^21,22^, polarization^23,24^, semantics such as edge^25^ or feature^26^, and so on. The higher imaging performance and additional information beyond intensity may open a new venue for subsequent intelligent processing, thus enabling challenging applications such as in-vivo deep-tissue imaging^27^, astronomical imaging^4^ non-line-of-sight imaging^28^, and so on.

**Fig. 2:**
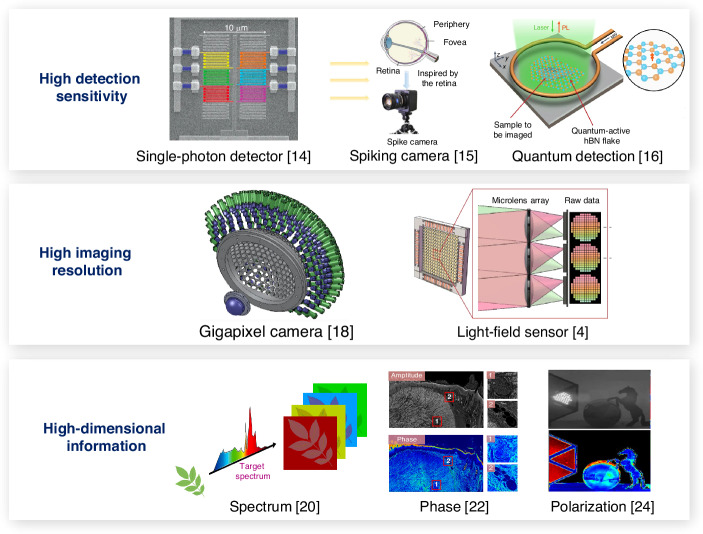
Overview of introducing novel detection techniques to enhance detection sensitivity, imaging resolution, and information dimension. The subfigures are cropped from corresponding references

One should note that although the introduced novel devices can indeed enhance imaging performance in certain aspects, “every coin has two sides.” In the event or spiking detection case, the fast imaging speed along with binary signals sacrifices bright field information that are more in line with human vision^15^. In high-resolution or high-dimensional detection situations, the increased data amount puts a heavier load on data transmission and post-processing. In this regard, one should consider each pros and cons for different specific applications, thus finding the most suitable elements to obtain the most needed imaging performance. On the other hand, introducing the multimodal fusion strategy^29,30^ can back up each other and alleviate the shortcomings of different devices.

Looking forward, the advancements in multiple fields including material science, integrated circuit, computer science, together with their interdisciplinarity, will boost the development of next-generation optical sources, elements, and detectors, leading to groundbreaking imaging techniques in not only microscopy but also mesoscopic and macroscopic detection^31,32^. Especially in the era of artificial intelligence and large models, imaging systems can further obtain intelligence to create new applications and revolutionize our observation and understanding of the natural world.
